# HK2 Mediated Glycolytic Metabolism in Mouse Photoreceptors Is Not Required to Cause Late Stage Age-Related Macular Degeneration-Like Pathologies

**DOI:** 10.3390/biom11060871

**Published:** 2021-06-11

**Authors:** Shun-Yun Cheng, Anneliese Malachi, Joris Cipi, Shan Ma, Richard S. Brush, Martin-Paul Agbaga, Claudio Punzo

**Affiliations:** 1 Department of Ophthalmology and Visual Sciences, University of Massachusetts Medical School, Worcester, MA 01655, USA; Shun-Yun.Cheng@umassmed.edu (S.-Y.C.); Anneliese.Malachi@umassmed.edu (A.M.); Joriscipi@gmail.com (J.C.); labmashan@gmail.com (S.M.); 2 Departments of Cell Biology and Ophthalmology and the Harold Hamm Diabetes Center, University of Oklahoma Health Sciences Center, Oklahoma City, OK 73104, USA; Richard-Brush@ouhsc.edu (R.S.B.); Martin-Paul-Agbaga@ouhsc.edu (M.-P.A.); 3 Dean A. McGee Eye Institute, University of Oklahoma Health Sciences Center, Oklahoma City, OK 73104, USA; 4 Horae Gene Therapy Center, University of Massachusetts Medical School, Worcester, MA 01605, USA; 5 Li Weibo Institute for Rare Diseases Research, University of Massachusetts Medical School, Worcester, MA 01605, USA

**Keywords:** AMD, aerobic glycolysis, glycolytic metabolism, photoreceptors, GA, CNV, wet AMD, dry AMD

## Abstract

Age-related macular degeneration (AMD) is a multifactorial disease of unclear etiology. We previously proposed that metabolic adaptations in photoreceptors (PRs) play a role in disease progression. We mimicked these metabolic adaptations in mouse PRs through deletion of the tuberous sclerosis complex (TSC) protein TSC1. Here, we confirm our previous findings by deletion of the other complex protein, namely TSC2, in rod photoreceptors. Similar to deletion of *Tsc1*, mice with deletion of *Tsc2* in rods develop AMD-like pathologies, including accumulation of apolipoproteins, migration of microglia, geographic atrophy, and neovascular pathologies. Subtle differences between the two mouse models, such as a significant increase in microglia activation with loss of *Tsc2*, were seen as well. To investigate the role of altered glucose metabolism in disease pathogenesis, we generated mice with simulation deletions of *Tsc2* and hexokinase-2 (*Hk2*) in rods. Although retinal lactate levels returned to normal in mice with *Tsc2-Hk2* deletion, AMD-like pathologies still developed. The data suggest that the metabolic adaptations in PRs that cause AMD-like pathologies are independent of HK2-mediated aerobic glycolysis.

## 1. Introduction

Age-related macular degeneration (AMD) is the main cause of blindness in the elderly [1,2]. The affected population is predicted to grow to 288 million people worldwide by 2040. The multifactorial nature of the disease and its still elusive etiology complicate the discovery of effective treatments. The only drugs available to date inhibit vascular endothelial growth factor (VEGF) function in patients with the exudative form of AMD that develop neovascular pathologies. This means that 85% of AMD patients who suffer from the other advanced form of the disease, namely geographic atrophy (GA), remain without treatment options [1].

Many studies have focused on identifying factors that contribute to disease progression [3]. During the early stages of the disease, lipid-rich extracellular deposits, known as drusen, form at the retinal pigment epithelium (RPE)–Bruch’s membrane (BrM) boundary and in the subretinal space [4,5]. Certain types of drusen deposits increase the risk of disease progression to the advanced stages of GA and/or choroidal neovascularization (CNV) [4,5]. Another major risk factor for progression to the advanced stages is a dysfunctional immune system [6]. Specifically, the dysregulation of complement factors, such as complement factor H (CFH) and complement component (C3), have been associated with progression to GA [7,8]. Dysregulation of complement factors can also cause inflammasome activation in the RPE and activation of microglia [9]. During disease progression, microglia can migrate to the subretinal space and release proinflammatory cytokines that contribute to CNV [9,10,11]. However, animal models based on these risk factors do not fully explain the disease etiology [12,13], suggesting that there are other unknown factors that participate in the development of AMD.

Our recent study proposed a model whereby an age-related metabolic shift in PRs constitutes an additional risk factor for disease progression [14]. During the early stages of AMD, the development of a hydrophobic lipoprotein wall at the BrM, which also gives rise to soft drusen [4,5], reduces the nutrient transfer of hydrophilic molecules such as glucose from the fenestrated choriocapillaris to the outer retinal cells, including PRs, creating a nutrient-deprived environment [14]. We proposed that similar to retinitis pigmentosa, the reduction in glucose experienced by PRs leads to a metabolic adaptation in PRs, in order to prolong their survival [15,16,17,18,19,20]. Indeed, we found two key glucose-related genes, HK2 and pyruvate kinase muscle isozyme M2 (PKM2), upregulated in PRs of AMD patients [14]. We showed that both genes play a pivotal role in the survival of nutrient-deprived cones in retinitis pigmentosa [15,16,17,18,19,20]. Additionally, we showed that the activity of the mammalian target of rapamycin complex 1 (mTORC1), a kinase that regulates cell metabolism by adapting supply with demand, is required to help cones to adapt to a nutrient shortage, and that further increasing mTORC1 activity not only increases the expression of *Hk2* and *Pkm2*, but also improves cone survival in retinitis pigmentosa [15,18,19,21]. To study the effect of the metabolic adaptions seen in PRs of AMD patients, we mimicked these adaptations by constitutive activation of mTORC1 in a non-diseased mouse retina [14]. This was sufficient to induce in wild-type mice AMD-like hallmarks, including the severe advanced forms of the disease such as GA and CNV [14]. Because these mice develop AMD in a progressive manner [14], the findings suggest that metabolic adaptations in PRs are a critical factor in the pathogenesis of AMD. What remains unclear is which of the metabolic alterations induced by activation of mTORC1 is responsible for the onset and progression of AMD-like pathologies in our mouse model.

Photoreceptors are among the most metabolically active cells in the human body [22]. They need large quantities of adenosine triphosphate (ATP) to maintain membrane potential, and nicotinamide adenine dinucleotide phosphate (NADPH) to recycle the visual chromophore [23,24] and to replenish the fatty acids lost during the shedding of the photoreceptor outer segments (POS) [25,26]. On a daily basis, a PR sheds 10% of its POS [27], which equates to one cell division per day in terms of lipid and protein content that needs to be replenished [28,29,30,31]. To fulfill their energy demands, PRs metabolize large quantities of glucose via aerobic glycolysis. This allows for a fast energy production, while also diverting enough glycolytic intermediates into the pentose phosphate shunt for NADPH synthesis. The lactate secreted by PRs is then used by Müller glia and RPE cells for energy production [32]. Both RPE and, to a certain extent, Müller glia are part of the retinal blood barrier that shields the retinal neurons from the choroidal and retinal vasculatures, respectively [33,34,35]. Thus, they are ideally suited to transfer glucose to retinal neurons, while relying themselves on PR-produced lactate. In addition, RPE cells also metabolize fatty acids from shed POS through β-oxidation [36,37,38,39]. This metabolic interdependence between the retina and the RPE creates a metabolic ecosystem geared to optimize the use of energy resources [32,40,41,42]. However, dysregulation of this ecosystem can also be detrimental to both retinal and RPE cells [32,41,42,43,44]. We showed that nutrient-deprived cone PRs upregulate the expression of genes related to glucose metabolism, such as glucose transporter 1 (*Glut1*), *Pkm2*, and *Hk2*, in order to take up more glucose and divert a higher percentage of the remaining glucose into the anabolic pathway. This helps maintain POS growth [15,16,17,18]. Others have proposed that the RPE starts to metabolize glucose in the absence of sufficient PR-derived lactate or fatty acids from POS [40]. Since constitutive activation of mTORC1 alters several aspects of cell metabolism including increasing aerobic glycolysis [45,46], and we showed that loss of *Tsc1* in PRs of wild-type mice leads to AMD-like pathologies [14], we asked whether changes in aerobic glycolysis that could affect the retinal-RPE metabolic ecosystem underlie the development of these pathologies. We focused this study on rods alone to reduce the complexity of the genetic crosses, since we have previously shown that deletion of *Tsc1* in cones, rods, or cones and rods together leads to the same overall pathologies by 12 months of age [14]. Anatomically, the human and mouse retinas are very similar, except for the absence of a fovea and macular pigment in mouse. Since AMD does not only affect foveal cones, and we showed that the overall pathologies are similar [14], the use of rods alone in our experimental setting serves as a good proxy to address the role of aerobic glycolysis in disease pathogenesis. Moreover, rods are more reliant on aerobic glycolysis than cones [15].

In this study, we confirm our previous findings, by altering PR metabolism through deletion of the TSC2 protein in rod PRs (referred to as ^r*od*^*Tsc2^−/−^* mice), by use of the *Cre-lox* system [47]. TSC1 and TSC2 are constituent proteins of the TSC complex that negatively regulates mTORC1 activity [46]. Similar to *^rod^Tsc1^−/−^* mice [14], *^rod^Tsc2^−/−^* mice develop hallmarks of both early- and late-stage AMD. Interestingly, we saw a higher frequency of microglia migration and reactivity in the subretinal space of *^rod^Tsc2^−/−^* mice, particularly microglia attached to the RPE surface, which was not apparent in *^rod^Tsc1^−/−^* mice. Moreover, GA developed either as seen in *^rod^Tsc1^−/−^* mice [14], or in a circular pattern, similar to what is seen in humans with GA. Finally, a higher glycolytic activity as a result of constitutively activated mTORC1 was also seen in *^rod^Tsc2^−/−^* mice, as assessed by increased retinal lactate levels. To determine whether PR aerobic glycolysis plays a role in AMD pathogenesis, we generated mice with deletion of *Tsc2* and *Hk2* in rods (referred to as *^rod^Tsc2^−/− rod^Hk2^−/−^* mice). Retinal lactate levels returned to normal in ^r*od*^*Tsc2^−/− rod^HK2^−/−^* mice; however, they still developed AMD-like phenotypes. The data indicate that metabolic adaptations in PRs that contribute to AMD pathologies are independent of *Hk2*-mediated aerobic glycolysis. This suggests that alterations in the glucose and lactate homeostasis between the PRs and the RPE are not what contributes to AMD pathologies in our mouse model.

## 2. Research Design and Methods

### 2.1. Animals

All experiments involving mice were in compliance with the Association for Research in Vision and Ophthalmology (ARVO) Statement for the Use of Animals in Ophthalmic and Vision Research. All procedures were approved by the Institutional Animal Care and Use Committees (IACUC) of the University of Massachusetts Medical School. Mice were kept on a 12 h light/12 h dark cycle with unrestricted access to diet and water. All mouse lines used (*Tsc2^c/c^* [48], *Raptor^c/c^* [49], *Hk2^c/c^* [50], *i75Cre* [51]) were described previously, and crossed as outlined in our recent publication [14]. The *i75Cre* is a rod PR-specific *Cre* line. The *Cre^+^* mice designation is synonymous with loss of function of a specific allele (e.g., *^rod^Tsc2^–/–^*, meaning loss of *Tsc2* in rod photoreceptors) while the *Cre^–^* control mice designation (e.g., *^rod^Tsc2^+/+^*) is synonymous with littermate controls emerging from a cross of a *Cre^+^* mouse and a *Cre^–^* mouse, both homozygous for the conditional allele in question. For the mice with the *Raptor* conditional allele, heterozygous (*Tsc2^C/C^ Raptor^C/+^*) and homozygous (*Tsc2^C/C^ Raptor^C/C^*) *Raptor* mice were crossed together with one of the two also carrying the *i75Cre* transgene to generate enough animals of the three genotypes presented in the data. These genotypes were then compared with mice with the *Tsc2^C/C^* that carry the *i75Cre* transgene (*^rod^Tsc2^–/–^*). Both male and female mice were used in this study. No sex-specific difference became apparent with any of the phenotypes described in this study. All mice were genotyped to confirm the absence of the confounding rd8 mutation in the *Crumbs1* gene [52].

### 2.2. Funduscopy, Angiography, and Optical Coherence Tomography (OCT)

Funduscopy, angiography, and OCT were performed as previously described [14]. In brief, mice received Phenylephrine (2.5%) and Tropicamide (1%) eye drops for dilation, and were then anesthetized with a mixture of ketamine/xylazine (100 mg/kg and 10 mg/kg). Funduscopy was performed first, followed by fundus fluorescein angiography (FFA). Fluorescein sodium solution (125 mg/kg) was injected subcutaneously in the neck region just before performing FFA. In cases where there was an apparent pathology by fundus imaging, OCT imaging was performed prior to fluorescein injection. Number and age of mice analyzed are indicated in the corresponding figures. Images were acquired with the Micron IV System from Phoenix Technology Group, which includes the OCT module.

### 2.3. Identification of Pathologies by Funduscopy for Quantification Purposes

Microglia pathology can be easily determined by funduscopy based on the appearance of small white foci on the fundus image. Paler foci tend to be microglia that are attached to the retina. An example of microglia on fundus images with corresponding RPE flat mount can be seen in the figure of Section 3.4, which shows 2 fundus images, one with many microglia (*^rod^Tsc2^−/− rod^Hk2^−/−^*) and one with none (*^rod^Tsc2^+/+ rod^Hk2^+/+^* mice). Their corresponding RPE flat mounts are shown in the same figure confirming the fundus images. Retinal folds appear as very bright dots that are much larger in size than microglia. The brightness is due to the fact that the fold funnels the light of the fundus machine. We have characterized these folds previously, and their characterization can be found in our previous study [14]. Neovascular pathologies were identified solely by FFA, with examples shown in the data of Section 3.1 and Section 3.3. The presence of GA was often confirmed on RPE flat mounts as shown in data of Section 3.1. On fundus, GA appears as a discoloration that is neither microglia or a fold. To determine our accuracy in properly assigning a fundus pathology as GA, we performed in our previous study 22 flat mounts of randomly selected eyes, 7 of which were assigned to the GA group by funduscopy [14]. Out of the 22 RPE flat mounts, 9 were confirmed to have GA, indicating that we may slightly underestimate the overall percentage of GA in our mouse population.

### 2.4. Electroretinography (ERG)

All ERG recordings were performed with the Celeris system (Diagnosys LLC) using the preset protocols for both scotopic and photopic ERG recordings. Data shown were recorded with the following parameters: Scotopic recordings were performed at 0.1 cd.s/m^2^. Photopic ERG recordings used a background intensity of 9 cd.s/m^2^ and a flash intensity of 10 cd.s/m^2^. Handling of the animals was performed similarly to funduscopy using the same anesthesia and dilation methods. Number of mice analyzed per group is indicated in the corresponding figures.

### 2.5. Histological Analyses

Immunofluorescence on either cryopreserved sections (12 µm thickness) or RPE flat mounts was performed as described previously [53]. The following primary antibodies were used: rabbit anti-ZO1 (1:100; Invitrogen, Cat#40-2200), rabbit anti-Iba1 (1:300; Wako, Cat#019-19741), and mouse anti-Rhodopsin (1:100, originally obtained from the University of British Columbia, Clone 1D4, available from Abcam, cat# 5417), all diluted in PBS with 0.3% Triton X-100 and 5% bovine serum albumin (BSA, Cell Signaling Technology). For the rabbit anti-Apolipoprotein B (ApoB) (1:800; Abcam, Cat# 20737), goat anti-Apolipoprotein E (ApoE) (1:1000, Millipore, Cat#178479), rabbit anti-CFH (1:300; Cat# ABIN3023097), and goat anti-mouse complement C3 (1:300; MP Biomedicals, cat# 55510), Triton X-100 was replaced with 0.2% Saponin. The following reagents already had a chromophore conjugated: fluorescein anti-mouse complement C3 (1:300; MP Biomedicals, cat# 55510), rhodamine phalloidin (1:1000; Life Technologies, Cat# R415), fluorescein peanut agglutinin lectin (PNA) (1:1000; Vector Laboratories, Cat# FL1071), and fluorescein Griffonia Simplicifonia Lectin I (GSL I) isolectin B4 (1:300; Vector Laboratories, Cat# FL-1201). Nuclei were counterstained with 4′, 6-diamidino-2-phenylindole (DAPI) (Sigma-Aldrich, Cat# 9542). All secondary antibodies (1:500, donkey) were purchased from Jackson Immuno Research and were purified F(ab)2 fragments that displayed minimal cross-reactivity with other species. Expression changes for ApoB, ApoE, C3, and CFH were confirmed in at least 3 individual animals per genotype. The genotypes of RPE flat mounts for microglia analyses were blinded prior to quantification with the LAS (Leica) software numbering function, to count the microglia by hand. All images were visualized with a Leica DM6 Thunder microscope with a 16-bit monochrome camera.

### 2.6. RPE Phagocytosis Activity Analysis

Quantification of POS clearance was performed similarly to other published literature [54] with the following modifications: Per RPE flat mount, 10 areas of 40,000 μm^2^ within a 1.5 mm radius from the center were selected randomly to quantify the number of RHODOPSIN positive dots per RPE cell. Images for quantification were acquired at 20×. RPE cell boundaries were detected with anti-ZO1 antibody. Quantification was performed using IMARIS imaging processor by selecting a dot diameter >2 μm to count dots and by counting the number of RPE cells per imaged field. The average dot number per RPE cell for a given RPE flat mount was obtained by averaging the results of the 10 fields. This number was then used to generate the average of the biological replicates (*n* = 6), per genotype and time point. All POS clearance experiments were performed with 2-month-old mice.

### 2.7. Lactate ASSAY

Lactate assay (L-Lactate Assay kit, Abcam, Cat# ab65330) was performed as previously described [14]. Two retinas from the same animal were pooled as a single biological sample. Each biological measurement was performed in duplicate. Each group contains 4–5 biological samples. Retinas were collected at 2 M and dissected in ice cold PBS. All retinas were flash frozen, stored at –80 °C, and processed all at once, according to the manufacturer’s instructions, after all samples were collected. Lactate measurements are shown as relative expression changes to littermate controls.

### 2.8. Quantitative Western Blot Analysis

All Western blot quantifications used 4 biological samples of 2-month-old mice with each sample consisting of both retinas from the same mouse. The analysis of each sample was performed in duplicate. Protein sample preparation and Western blot analysis were performed with the same reagents and techniques as previously described [14]. In brief, enucleated eyes were dissected in cold PBS buffer. Dissected retinas were immediately transferred into RIPA buffer (Thermo Scientific, cat# 89900) with protease and phosphatase inhibitors (1:100 dilution; cat#1861281) and homogenized by sonication. After 10 min centrifugation at 4 °C at 13,000 RPM, protein extracts were transferred into a fresh tube and protein concentration was quantified with the Bio-Rad Protein Assay (cat# 500-0113, 0114, 0115). To quantify PKM2 and p-S6 expression levels, 5 μg and 10 μg of total protein, respectively, were loaded. The following primary antibodies from Cell Signaling Technology were used: rabbit anti-PKM2 antibody (1:4000; Cat#4053), rabbit anti-pS6 (Ser240/244) (1:1000; Cat#5364), and for normalization, mouse anti-β-actin antibody (1:1000; Cat#3700). Protein detection was done using fluorescently labeled secondary (1:10,000) antibodies from Licor in combination with the Odyssey system. Quantification was performed with Image Studio software.

### 2.9. Lipid Profiling

Lipid profiling was performed with 2-month-old mice. Each biological sample (*n* = 3) consists of two retinas from the same animal. The analytical methods have been described previously [55]. Briefly, tissue was homogenized in 40% aqueous methanol and then diluted to a concentration of 1:40 with 2-propanol/methanol/chloroform (4:2:1 v/v/vol) containing 20 mM ammonium formate and 1.0 μM PC (14:0/14:0), 1.0 μM PE (14:0/14:0), and 0.33 μM PS (14:0/14:0) as internal standards. Samples were introduced into a triple-quadrupole mass spectrometer (TSQ Ultra, Thermo Scientific) by using a chip-based nano-ESI source (Advion NanoMate) operating in infusion mode. PC lipids were measured using precursor ion scanning of m/z184. PE lipids were measured using neutral loss scanning of m/z141. All species detected for each group are represented as a relative percentage of the sum based on their response values. Abundances of lipid molecular species were calculated using the Lipid Mass Spectrum Analysis (LIMSA) software (University of Helsinki, Helsinki, Finland).

### 2.10. Statistical Analysis

Multiple *t*-test was used for two-group comparisons, and two-way ANOVA for comparisons of more than two groups. Both analysis types were two-tailed. Significance levels: * *p* < 0.05; ** *p* < 0.01; *** *p* < 0.001; **** *p* < 0.0001. All bar graphs indicate mean, and error bars represent standard error of the mean (S.E.M.). Fundus analysis bar graphs show the percentage of mice that developed the retinal pathologies described, while error bars represent margin of errors (M.O.E.) calculated with a 90% confidence interval.

## 3. Results

### 3.1. ^rod^Tsc2^−/−^ Mice Develop AMD-Like Pathologies Similar to ^rod^Tsc1^−/−^ Mice

**Figure 1:**
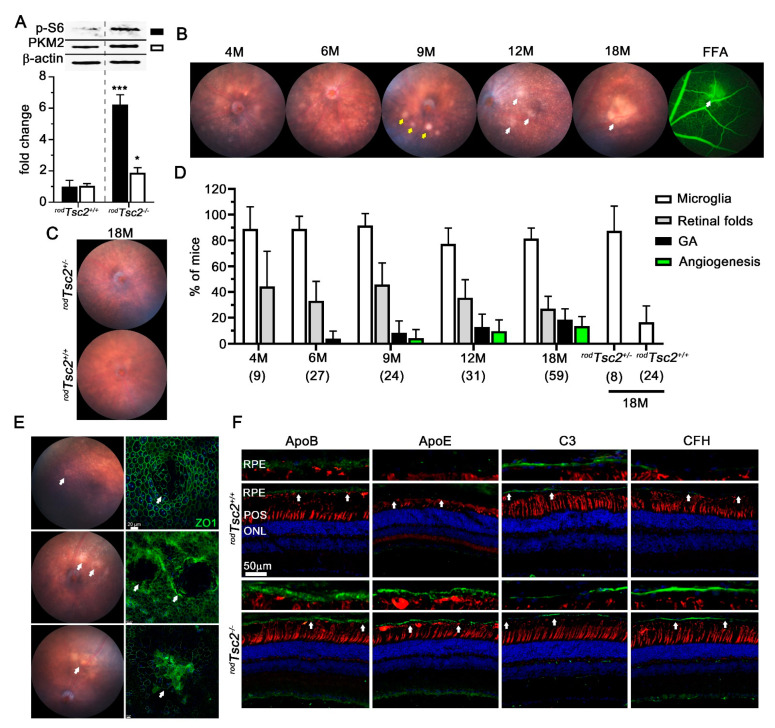
*^rod^Tsc2^−/−^* mice develop AMD-like pathologies. (**A**) Western blot quantifications performed with 2 months (M) old mice for p-S6 (black bar) and PKM2 (white bar) expression levels in *^rod^Tsc2^+/+^* and *^rod^Tsc2^−/−^* mice (*n* = 4 mice). On top are representative Western blot images for p-S6 (32kDa), PKM2 (60kDa), and the β-Actin control (45kDa). Relative fold changes are shown. Error bars: S.E.M., * *p* < 0.05; *** *p* < 0.001. (**B**) Fundus and fundus fluorescein angiography (FFA: right) images of *^rod^Tsc2^−/−^* mice over time (yellow arrows: retinal folds; white arrows: GA or neovascular pathology in FFA image; prominent white dots on fundus image of 12 M are microglia). (**C**) Representative fundus images of *^rod^Tsc2^+/−^* (top) and *^rod^Tsc2^+/+^* (bottom) mice at 18 M. (**D**) Quantification of pathologies seen by funduscopy and FFA in *^rod^Tsc2^−/−^* mice over time and littermate controls at 18 M. Each bar shows percentage of mice ± M.O.E. Numbers in parentheses represent number of mice analyzed per time point. (**E**) Fundus (left) and RPE flat mount (right; ZO-1: Zona Occludens 1 protein marking tight junctions of RPE cell boundary in green) images showing the development of the different GA types seen in *^rod^Tsc2^−/−^* mice at 12 M. Slow intermediate GA (top), severe circular formation of GA (middle), and irregular patchy form of GA (bottom) (white arrows: GA lesions). (**F**) Immunofluorescent images of retinal cross-sections from *^rod^Tsc2^+/+^* (top) and *^rod^Tsc2^−/−^* (bottom) mice at 12 M of age showing different AMD-related markers in green: ApoB, ApoE, C3, CFH. Higher magnification of RPE–BrM area marked between the 2 white arrows is shown above each section (blue: DAPI; red: PNA marking cone segments; scale bar = 50 μm).

We previously showed that *^rod^Tsc1^−/−^* mice develop AMD-like pathologies that are dependent on the activity of mTORC1. To further confirm our previous findings and exclude any unknown mechanism that might be associated with loss of the TSC1 protein itself, we deleted the other TSC complex component, namely *Tsc2*, in mouse rods (*^rod^Tsc2*^−*/*−^), by use of the *Cre-lox* system [47], and evaluated its effect on disease development. Increased mTORC1 activity in *^rod^Tsc2*^−*/*−^ retinas was verified by Western blot analysis for changes in phosphorylation of ribosomal protein S6 (p-S6), a bonafide mTORC1 downstream target. Changes in aerobic glycolysis were assessed by measuring PKM2 protein levels and retinal lactated levels (lactate levels are shown together with *Hk2* knockout data in Section 3.3), since *Hk2*, as a glycolytic gene, has already been shown to be regulated directly by mTORC1 activity [14,45,46]. We found a 6-fold increase in p-S6 levels, a 1.8-fold increase in PKM2 levels, and a 1.2-fold increase in retinal lactate levels in *^rod^Tsc2*^−*/*−^ mice, when compared with their littermate controls (Figure 1A), indicating that mTORC1 activity was increased and aerobic glycolysis was altered.

To examine whether *^rod^Tsc2*^−*/*−^ mice develop AMD-like pathologies, we conducted funduscopy and fundus fluorescein angiography (FFA) analyses over a period of 18 months (M) (Figure 1B–D). By 4 M, 90% of mice had microglia migrating into the subretinal space. Microglia appear as small white foci on the funduscopy images. They remained abundant as mice aged (Figure 1D). A more detailed characterization of the microglia migration is described in Section 3.4. The formation of retinal folds was also observed in some *^rod^Tsc2*^−*/*−^ mice. However, the frequency was lower than that seen in our previous study (Figure 1D) [14]. GA was seen in 2% of mice at 6 M and 23% of mice at 18 M (Figure 1D), while neovascular pathologies, as assessed by FFA, were seen in 18% of mice at 18 M of age (Figure 1B,D). Similar to our previous findings [14], heterozygous *^rod^Tsc2^+/−^* mice also showed a propensity to accumulate microglia when compared with their age-matched littermate controls (Figure 1C,D). However, neither aged control (*^rod^Tsc2^+/+^*) mice nor heterozygous *^rod^Tsc2^+/−^* mice developed any advanced AMD-like pathologies (Figure 1C,D), confirming the dose-dependent effect observed in our previous study [14]. Interestingly, *^rod^Tsc2^−/−^* mice exhibited a more diverse pattern of GA, even though GA developed slower, when compared with the *^rod^Tsc1^−/−^* mice. Unlike *^rod^Tsc1^−/−^* mice, *^rod^Tsc2^−/−^* mice also developed a form of GA that is characterized by a very distinctive boundary that expands circularly, similar to what is seen in humans (Figure 1E, top and middle). This form of GA appeared to be slow progressing. RPE flat mounts of most mice were found at the stage shown in the top panel of Figure 1E, with atrophic RPE cells in the center and hypertrophic RPE cells surrounding the center (Figure 1E, top). Only a few RPE flat mounts showed complete RPE atrophy in the center (Figure 1E, middle). The other form of GA that was seen is similar to that described previously [14], with irregular hypertrophic and atrophic RPE cells (Figure 1E, bottom) that then collapse to form an area of complete RPE loss. Finally, to confirm that *^rod^Tsc2^−/−^* mice also develop early AMD disease hallmarks, we analyzed expression changes of apolipoproteins B and E, at the RPE–BrM, as well as CFH and C3. Paralleling our previous findings [14], *^rod^Tsc2*^−*/*−^ mice accumulate ApoB and ApoE as well as CFH and have reduced expression of C3 (Figure 1F). These changes were uniform across the entire eye regardless of the presence of the GA or any neovascular pathology. Together, the data confirm our previous findings [10] indicating that activation of mTORC1 in PRs is sufficient to cause AMD-like pathologies in mice reminiscent of those seen in patients with AMD.

**Figure 2:**
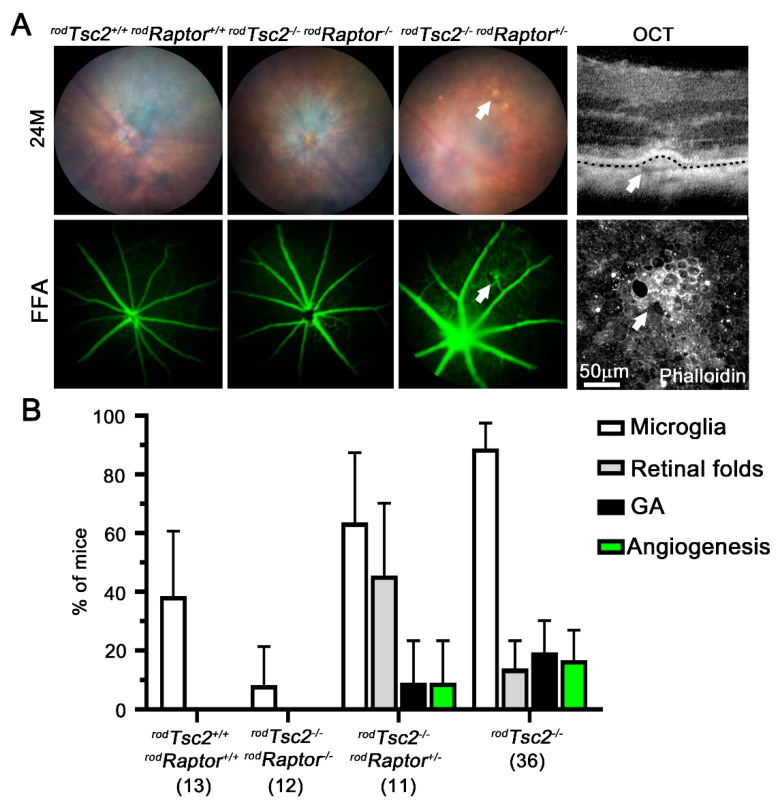
mTORC1 activity in PRs is required for *^rod^Tsc2^−/−^* mice to develop pathologies. (**A**) Representative fundus and FFA images of *^rod^Tsc2^+/+rod^Raptor^+/+^*, *^rod^Tsc2^−/−rod^Raptor^−/−^*, and *^rod^Tsc2^−/−rod^Raptor^+/−^* mice at 24 M of age. Far right column shows OCT (top) image, and corresponding RPE flat mount (bottom) of *^rod^Tsc2^−/−rod^Raptor^+/−^* fundus image is shown in the third column, where the beginning of a mild GA and neovascular pathology is seen (arrows). (**B**) Percentage distribution of pathologies seen at 24 M by funduscopy and FFA, in genotypes indicated. Each bar shows percentage of mice ± M.O.E. Number in parentheses represent number of mice analyzed per genotype.

To confirm that mTORC1 activity was also required in mice with loss of *Tsc2* in rods (*^rod^Tsc2^−/−^*) to cause AMD-like pathologies, we generated mice with simultaneous deletion of *Tsc2* and the mTORC1 accessory protein Raptor (*^rod^Tsc2^−/− rod^Raptor^−/−^* mice) (Figure 2). At 24 M of age, none of the *^rod^Tsc2^−/− rod^Raptor^−/−^* mice developed any advanced AMD-like pathologies except for the accumulation of microglia, which was also seen in age-matched wildtype control mice. Interestingly, in heterozygous *Raptor* knockout mice, *^rod^Tsc2^−/− rod^Raptor^+/−^* mice, 1 out of 11 mice developed GA and a neovascular pathology (Figure 2A,B). Optical coherence tomography (OCT) imaging showed a hyperreflective RPE detachment from the BrM, resembling an edema that overlapped with the neovascular pathology, as assessed by fundus fluorescein angiography (FFA). RPE flat mount analyses of the area showed a hyperfluorescent dysmorphic RPE, indicative of an early progression to atrophy. In age-matched *^rod^Tsc2^−/−^* mice, the frequency of GA and neovascularization was 20% and 18%, respectively, which is similar to what was seen in *^rod^Tsc2^−/−^* mice at 18 months (Figure 2B). Together, the data confirm that mTORC1 activity is required to cause advanced AMD-like pathologies in a dose-dependent manner.

### 3.2. RPE Phagocytosis Activity Is Disrupted in ^rod^Tsc2^−/−^ Mice

**Figure 3:**
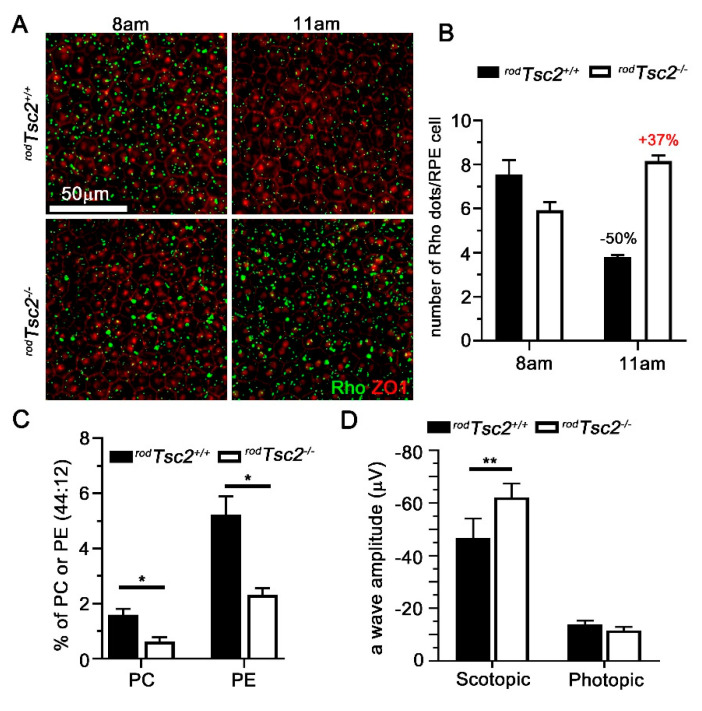
RPE phagocytic activity is delayed in 2 M old *^rod^Tsc2^−/−^* mice. (**A**) Representative images used for quantification in (**B**) of RPE flat mounts at 8am and 11am, showing accumulation of shed POS in both *^rod^Tsc2^+/+^* and *^rod^Tsc2^−/−^* mice at 2 M (green: Rhodopsin; red: ZO-1, Zona Occludens 1 protein marking tight junctions of RPE cell boundary; scale bar = 50 μm). (**B**) Quantification of number of POS (Rhodispins: RHO+ dots) accumulated on RPE flat mounts at 8am and 11am in 2 M old mice. Bar shows average number of POS (Rho) per RPE cell ± S.E.M. (*n* = 6 RPE flat mounts). (**C**) Percentage of di-DHA (44:12) PC and PE phospholipids in total retinal extracts from both *^rod^Tsc2^+/+^* (black bar) and *^rod^Tsc2^−/−^* (white bar) mice ± S.E.M. (*n* = 3 mice, * *p* < 0.05). (**D**) Average of scotopic and photopic ERG recordings from both *^rod^Tsc2^+/+^* (black bar) and *^rod^Tsc2^−/−^* (white bar) mice at 2 M of age. Bars show average a-wave amplitude (μV) ±S.E.M. (*n* = 8 and 14 mice, respectively; ** *p* < 0.01). Scotopic recordings were performed at 0.1 cd.s/m^2^. Photopic ERG recordings used a background intensity of 9 cd.s/m^2^ and a flash intensity of 10 cd.s/m^2^.

RPE cells phagocytose POS to release oxidative stress in PRs, while recycling at the same time fatty acids such as DHA from POS, to support their own nutrient needs. In *^rod^Tsc1^−/−^* mice, we found that constitutively activated mTORC1 in PRs results in a significant decrease in di-DHA (44:12, refers to 2 DHA side chains on the phospholipid) containing phosphatidylethanolamine (PE) (WT: 15%; *^rod^Tsc1^−/−^*: 5%) and phosphatidylcholine (PC) (WT: 5%; *^rod^Tsc1^−/−^*: 1%) lipids in total retinal lipid preparation. The reduction of these two types of phospholipids in POS correlated with a delay of POS clearance by the RPE in *^rod^Tsc1^−/−^* mice. We therefore inquired if there is also a delay in POS clearance in *^rod^Tsc2^−/−^* mice (Figure 3A,B). Because the peak POS shedding and RPE phagocytose is just after light onset (7am), we collected RPE flat mounts at 8am and 11am and calculated the percentage change in rhodopsin-positive POS/RPE cell detected between these two time points. We found at 2 M of age, a time point prior to any detectable pathologies by fundus, a similar increase in the percentage of remaining POS at 11am, as seen previously [14], indicating that POS clearance is also delayed in *^rod^Tsc2^−/−^* mice. In control *^rod^Tsc2^+/+^* mice, rhodopsin-positive dots decreased by 50% at 11am, while they increased by 37% in *^rod^Tsc2^−/−^* mice (Figure 3A,B). Consistent with this finding, there was a similar decline in di-DHA containing PE and PC lipids (Figure 3C). To determine if PR function was also affected, as seen previously [14], we performed electroretinogram (ERG) recordings at 2 M of age. Similar to our published data [14], there was a slight increase in the scotopic ERG a-wave response in *^rod^Tsc2^−/−^* mice (Figure 3D). The underlying cause of this increase remains unclear. It could be caused by higher energy availability, increased PKM2 expression, and/or overall increased mTORC1 activity [15,56,57].

### 3.3. HK2-Mediated Aerobic Glycolysis Is Not Required for Severe AMD Pathologies to Develop

**Figure 4:**
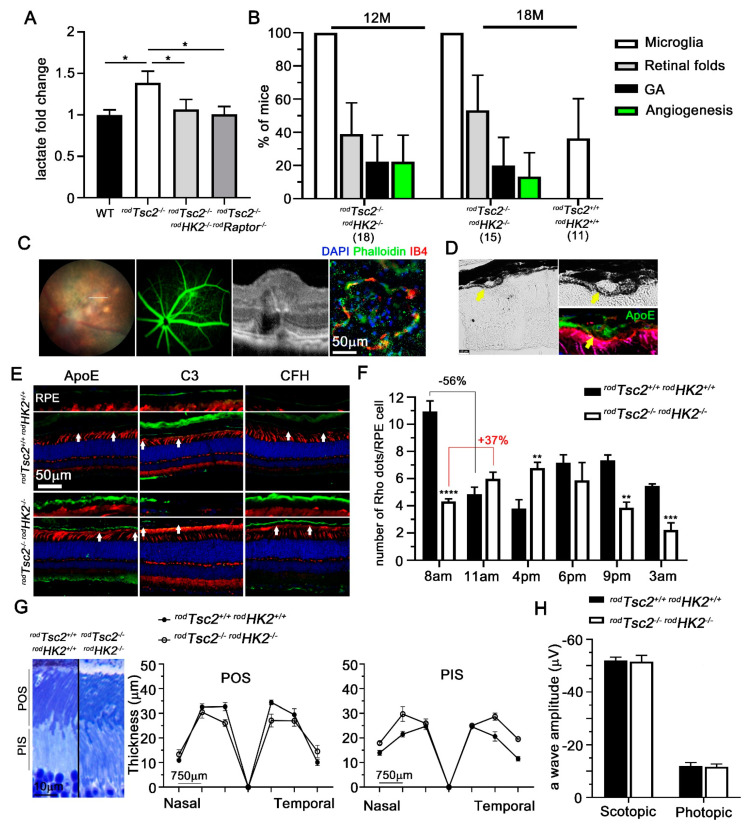
HK2-dependent aerobic glycolysis in PRs is not required for disease development. (**A**) Retinal lactate measurements from total retinal extracts at 2 M of age of genotypes indicated. Each bar shows relative fold change compared with each genotype’s littermate control ±S.E.M. (*n* = 4–6 mice, * *p* < 0.05). (**B**) Quantification of pathologies seen by funduscopy and angiography in *^rod^Tsc2^−/− rod^Hk2^−/−^* mice at 12 M and 18 M of age, and in littermate controls at 18 M of age. Each bar shows percentage of mice ± M.O.E. Number in parentheses represents number of mice analyzed per time point and genotype. (**C**) Analysis of the same GA and CNV lesion seen in *^rod^Tsc2^−/− rod^Hk2^−/−^* mice by fundus, FFA, OCT, and on RPE flat mount (far right). Line in first panel shows region of OCT scan. IB4 staining (red signal) on RPE flat mount marks blood vessels that have protruded through the BrM and RPE layer, while phalloidin marks remaining RPE cells. (**D**) Representative drusen-like deposit in *^rod^Tsc2^−/− rod^Hk2^−/−^* that also stains positive for ApoE (ApoE: green; phalloidin: red, marking RPE cell boundaries; PNA: magenta, marking cone segments). (**E**) Immunofluorescence images of *^rod^Tsc2^+/+ rod^Hk2^+/+^* (top) and *^rod^Tsc2^−/− rod^Hk2^−/−^* (bottom) mice at 12 M of age showing different AMD-related markers (green): ApoE, C3, CFH, as shown in Figure 1F. Higher magnification of RPE–BrM area (between 2 white arrows) is shown above each retinal section image (blue: DAPI; red: PNA; scale bar = 50 μm). (**F**) Quantification of number of POS (RHO+ dots) accumulated on RPE flat mounts at different time points during the day. Bars show average number of POS (Rho) per RPE cell ± S.E.M. (*n* = 6 RPE flat mounts per time point, ** *p* < 0.01, *** *p* < 0.001, **** *p* < 0.0001). (**G**) Measurements of POS length in *^rod^Tsc2^+/+ rod^Hk2^+/+^* and *^rod^Tsc2^−/− rod^Hk2^−/−^* mice. To the left: representative image of POS and photoreceptor inner segments (PIS) of the two genotypes. To the right: quantifications of relative length at 750 μm intervals from the optic nerve head in a nasal–temporal direction (value “0” represents optic nerve head). (**H**) Scotopic and photopic ERG measurement of both *^rod^Tsc2^+/+ rod^Hk2^+/+^* (black bar) and *^rod^Tsc2^−/− rod^Hk2^−/−^* (white bar) mice. Bars show average a-wave amplitude (μV) ±S.E.M. (*n* = 9 and 11 mice, respectively). Scotopic recordings were performed at 0.1 cd.s/m^2^. Photopic ERG recordings used a background intensity of 9 cd.s/m^2^ and a flash intensity of 10 cd.s/m^2^.

We have previously shown that activation of mTORC1 in wild-type rods increases retinal lactate and NADPH levels and causes AMD-like pathologies, while loss of HK2 decreases retinal lactate and NADPH levels [14,15]. Because of the intricate metabolic eco-system between the retina and the RPE, whereby glucose is transferred from the RPE to PRs and lactate from PRs to the RPE, changes in retinal glucose consumption and lactate production could be a contributing factor to the AMD-like pathologies seen in our mouse model. To elucidate if changes in glucose metabolism caused by activation of mTORC1 contribute to disease, we generated mice with a simultaneous deletion of *Tsc2* and *Hk2* in rods (*^rod^Tsc2^−/− rod^Hk2^−/−^*). To confirm that aerobic glycolysis was altered, we measured retinal lactate level in *^rod^Tsc2^−/− rod^Hk2^−/−^* mice at 2 M of age, before the development of any disease. In *^rod^Tsc2^−/−^* mice, there was a 1.2-fold increase in lactate production. In control *^rod^Tsc2^−/− rod^Raptor^−/−^* mice, where the increase in mTORC1 activity is prevented, the increase in lactate production did not occur (Figure 4A). Similarly, in *^rod^Tsc2^−/− rod^Hk2^−/−^* mice there was no increase in retinal lactate levels. Interestingly, the levels did not drop below their littermate control levels, even though removal of *Hk2* alone leads to a drop in retinal lactate production [15]. This could be due to other genes such as PKM2 that in the context of constitutively activated mTORC1 contribute to higher lactate levels [56]. Next, to determine if AMD-like pathologies still develop in aged *^rod^Tsc2^−/− rod^Hk2^−/−^* mice, we performed funduscopic analyses at 12 M and 18 M (Figure 4B). We found that at both time points, GA and neovascular pathologies occur at a similar rate to what is seen in *^rod^Tsc2^−/−^* mice (Figure 4B). Age-matched *Cre^–^* control mice only showed accumulation of microglia in a small percentage of mice (Figure 4B). Neovascularization in *^rod^Tsc2^−/− rod^Hk2^−/−^* mice, as detected by FFA, was further confirmed by OCT imaging and immunofluorescent staining on RPE flat mounts, also identifying choroidal neovascular lesions (Figure 4C). Early AMD hallmarks such as ApoE-positive drusen-like deposits were also seen in *^rod^Tsc2^−/− rod^Hk2^−/−^* mice (Figure 4D). Additionally, uniform accumulations of ApoE and CFH, and downregulation of C3 at the BrM–RPE interphase occurred as well in *^rod^Tsc2^−/− rod^Hk2^−/−^* mice (Figure 4E). Finally, RPE phagocytic activity was also perturbed (Figure 4F). Interestingly, the number of POS per RPE cell in *^rod^Tsc2^−/− rod^Hk2^−/−^* mice was much lower than in the *Cre^–^* littermate control group during peak of shedding (8am) (Figure 4F). To determine if there was a shift in the circadian clock, resulting in a delayed peak of shedding [25] in *^rod^Tsc2^−/− rod^Hk2^−/−^* mice, we repeated the experiment at different time points over a 24 h time period (Figure 4F). We found a small but continuous increase between 8am and 4pm, and then a continued decline over time. The data suggest that not only is there a delay in POS clearance, but also reduced overall POS shedding in mice with loss of *Hk2*. Measurements of POS length did not reveal any significant difference between the *Cre^–^* and the *Cre^+^* mice (Figure 4G). To determine if PR function was affected, we performed ERG recordings. Loss of *Hk2* in the context of constitutively activated mTORC1 reduced the scotopic ERG levels back to those of control littermate mice (Figure 4H). While this differs from loss of *Hk2* alone, where we observed a decline in the scotopic a-wave [15], the data align with the lactate measurements, where we found that levels dropped back to the levels seen in littermate control mice, rather than those seen with loss of *Hk2* alone [15]. In summary, the presence of early as well as late disease hallmarks in *^rod^Tsc2^−/− rod^Hk2^−/−^* mice, together with a delay in POS clearance, indicate that the development of AMD-like pathologies driven by metabolic changes in PRs is independent of a *Hk2*-mediated shift in aerobic glycolysis.

### 3.4. Microglia Migration and Reactivity Seen in the Subretinal Space

**Figure 5:**
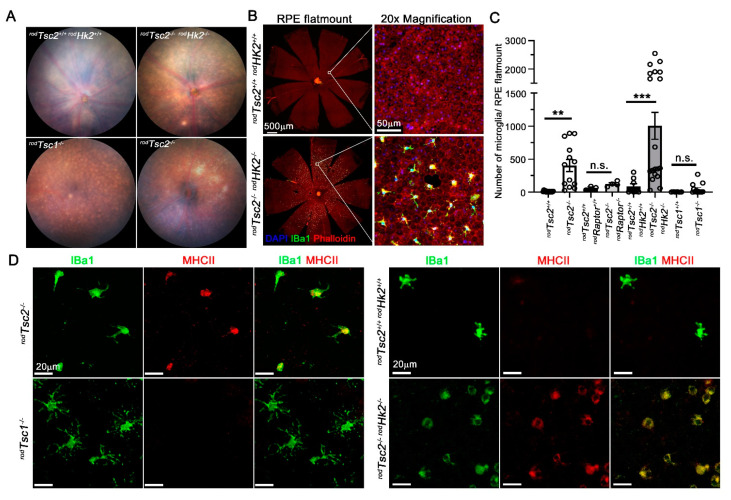
Microglia migration and activation seen in the subretinal space of 12 M old mice. (**A**) Representative fundus images of genotype indicated. For the *Cre^–^* negative controls, only the *^rod^Tsc2^+/+ rod^Hk2^+/+^* mice are shown. Quantification of all *Cre^–^* controls is shown in (**C**). (**B**) Representative RPE flat mounts and zoomed in images of two of the genotypes shown in (**A**), namely *^rod^Tsc2^−/− rod^Hk2^−/−^* mice and their *Cre^–^* controls, showing a large number of IBa1 positive (green) cells in *^rod^Tsc2^−/− rod^Hk2^−/−^* mice. (**C**) Quantification of total number of IBa1+ cells on RPE flat mounts of genotypes indicated at 12 M. Each bar shows the average number of IBa1+ cells accumulated on RPE flat mounts ±S.E.M. (Each dot shows cell count of a single RPE flat mount. Number of flat mounts per genotype varies between 4 and 20. Lower number was used for genotypes where microglia were rarely seen by fundus. ** *p* < 0.01; *** *p* < 0.001.) (**D**) Representative images of the two different types of IBa1+ (green) cells seen on RPE flat mounts that differ by their amount of MHCII signal (red). In *^rod^Tsc2^−/−^* mice and *^rod^Tsc2^−/− rod^Hk2^−/−^* mice, many IBa1+ cells have higher levels of MHCII expression. In *^rod^Tsc1^−/−^* mice, the IBa1-positive cells look more ramified with very low levels of MHCII (scale bar = 20 μm).

Several clinical studies report an abundance of microglia in the subretinal space of patients with AMD [58,59]. In both *^rod^Tsc2^−/−^* mice and *^rod^Tsc2^−/− rod^HK2^−/−^* mice, we found many small pronounced foci across the entire fundus, in much larger numbers than was observed in *^rod^Tsc1^−/−^* mice (Figure 5A). To further characterize this difference, we quantified the accumulation of microglia on RPE flat mounts (Figure 5B,C), using an Iba1 antibody to identify microglia. As expected, we found a small amount of Iba1+ cells on RPE flat mounts of aged *Cre^–^* control mice, as well as mice in which mTORC1 activity was abolished in the context of TSC2 loss (*^rod^Tsc2^−/− rod^Raptor^−/−^*) (Figure 5C). In both *^rod^Tsc2^−/−^* mice and *^rod^Tsc2^−/− rod^HK2^−/−^* mice, the number of microglia accumulated onto the RPE layer was significantly larger than the number seen in their age-matched littermate controls and than the number found in *^rod^Tsc1^−/−^* mice (Figure 5C), confirming the findings by funduscopy. Microglia reactivity can be detected by the different morphology of microglia and the expression levels of proteins such as MHCII. Compared with resident microglia, activated microglia display upregulation of MHCII and retraction of their processes from a ramified to an amoeboid morphology. Indeed, we observed two types of Iba1+ cells in *^rod^Tsc2^−/−^* mice and *^rod^Tsc2^−/− rod^Hk2^−/−^* mice (Figure 5D). One type displays a more ramified morphology with low expression levels of MHCII, while the other type, which appeared more abundant, displays an amoeboid morphology and high MHCII expression levels (Figure 5D). The small number of microglia seen in *^rod^Tsc1^−/−^* mice predominantly had a ramified morphology with low MHCII expression levels (Figure 5D). Similarly, the small number of Iba1+ cells found on the RPE in age-matched controls, and *^rod^Tsc2^−/− rod^Raptor^−/−^* mice, were also mostly ramified resident microglia with low MHCII expression levels (Figure 5D). The data suggest a possible role for PRs in contributing to microglia migration and activation through secreted signals.

## 4. Discussion

Age-related macular degeneration is the leading cause of blindness among the elderly. The underlying cause that results in disease remains unclear. Here, we show that loss of the second TSC complex component, *Tsc2*, in mouse PRs recapitulates the AMD-like pathologies seen in *^rod^Tsc1^−/−^* mice, albeit with slightly different kinetics. *^rod^Tsc2^−/−^* mice show a higher frequency of angiogenesis and a more robust migration of microglia into the subretinal space. We also observed a distinctive different pattern of GA from that seen in *^rod^Tsc1^−/−^* mice. In addition to the irregularly shaped areas of GA, we also saw regions of GA that displayed a clear circular boundary. Subtle differences between loss of *Tsc1* and *Tcs2* have been seen in many other studies, including in humans with hamartomas [60,61,62], which are benign tumors that are caused by loss of either gene. There are functions of the two proteins that are independent of the regulation of mTORC1 [63,64,65] that could modulate the disease progression in our mouse model. Some of these functions could affect the activity of AMD risk genes [3,66]. However, our experiments in this study and in our previous study [14] show that mTORC1 activity is required in our mouse model for pathologies to develop.

To further dissect possible mechanisms downstream of mTORC1 that contribute to disease progression in our mouse model, we generated mice with simultaneous deletion of *Hk2* and *Tsc2*. This corrected the increase in retinal lactate levels seen after constitutive activation of mTORC1; however, it did not prevent the occurrence of AMD-like pathologies. The data indicate that *Hk2*-mediated aerobic glycolysis changes do not contribute to AMD pathologies in our model. This suggests that changes in the glucose-lactate exchange between the retina and the RPE are unlikely to be the cause of the development of AMD. This does not mean that changes in the glucose-lactate exchange are not responsible for causing other adaptive gene expression changes in PRs that then contribute to the development of AMD.

Our mouse model was based on the premise that there is a glucose shortage in PRs of AMD patients. This premise was based on the increased expression of key metabolic genes, such as PKM2 and HK2, seen in PRs of AMD patients and other data from the literature that suggest that PRs are glucose-deprived and that their metabolism contributes to AMD [4,5,40,67,68,69]. For example, the buildup of lipids at the BrM suggests that glucose transfer from the choroidal vasculature to PRs should be reduced in patients with AMD [4,5]. By mimicking the adaptive response to the glucose shortage through constitutive activation of mTORC1, we likely altered several metabolic pathways, such as glycolysis, the pentose phosphate pathway, lipid synthesis, autophagy, and overall protein synthesis. Here, we establish that among these pathways, changes in glycolysis that are controlled by *Hk2* are unlikely to be the underlying cause of disease upon constitutive activation of mTORC1. While changes in glycolysis, due to a shortage of glucose, may be the driving force in humans for induction of the metabolic shift in PRs of AMD patients, this shift, which likely increases mTORC1 activity, may cause disease by one of the other pathways that is affected by increased mTORC1 activity.

In *^rod^Tsc2^−/−^* mice, we observed a higher frequency of neovascular pathologies and activation of microglia. While this may be a strain background difference between the *^rod^Tsc1^−/−^* and *^rod^Tsc2^−/−^* mice, it is also possible that TSC2 plays a role in mediating vascularization and microglia activation that is independent of mTORC1 activity. In brain cells, removal of *Tsc2* increases the expression of both mTORC1-dependent and -independent stress response genes, including hypoxia-inducible factor 1-alpha (Hif1a) [65]. HIF1a is a transcription factor that induces vascular endothelial growth factor (VEGF) expression under hypoxic conditions. While accumulation of HIF1a protein is an mTORC1-dependent event, loss of *Tsc2* still results in some increase in VEGF expression even when mTORC1 function is inhibited [65]. Since higher levels of VEGF can increase angiogenesis and also act as a chemotactic factor for microglia, the observations made in *^rod^Tsc2^−/−^* mice might be due to mTORC1-independent functions of TSC2 [65,70,71,72]. A clinical study has shown that patients who develop CNV have significantly lower expression levels in PRs of the soluble form of the VEGF receptor (sFLT-1) [73,74], attributing a potential role to PRs in maintaining VEGF homeostasis in AMD, and contributing to neovascular pathologies. Alternatively, strain background differences between the two strains could also account for these observations. Further investigations are required to elucidate the mechanism in *^rod^Tsc2^−/−^* mice that increases microglia activation and neovascular pathologies.

A surprising observation was that *^rod^Tsc2^−/−rod^HK2^−/−^* mice displayed a lower rate of POS shedding at 8am than their *Cre^–^* littermate control mice. This suggests that the observation is unlikely to be due to a strain background difference, but rather to the loss of HK2 itself, since *Cre^–^* littermate controls and *^rod^Tsc2^−/−^* mice show a similar rate of shedding at 8am. Since POS length was not significantly different between *^rod^Tsc2^−/−rod^HK2^−/−^* and their *Cre^–^* littermates, it would suggest that growth of the POS is slowed in *^rod^Tsc2^−/−rod^HK2^−/−^* mice and that shedding is a function of the time it takes the shed POS to reach the RPE microvilli, to initiate the next round of shedding. The difference in the PR inner segment length seen could be due to a larger number of mitochondria in mice with loss of *Hk2* [15] and/or to a general hypertrophy of the cytoplasm that is often seen with disruption of the TSC complex [75].

In summary, we show that disruption of the TSC complex in rods leads to AMD-like pathologies, irrespectively of the complex component that is removed, and that removal of *Hk2* does not prevent the development of these pathologies. The data suggest that the development of AMD-like pathologies in our mouse model is not driven by changes in the glucose-lactate exchange between the retina and the RPE. The changes downstream of mTORC1 that contribute to disease remain to be investigated. Identifying the major contributing factor downstream of mTORC1 that leads to disease might allow for the inhibition of such a factor, while still allowing PRs to adapt their metabolism to the nutrient shortage they experience in AMD patients. This would circumvent the problem of directly inhibiting mTORC1 activity in AMD patients, as has already been done in several studies [76,77] with no benefits or even negative outcomes for patients.

## Data Availability

All data and experimental parameters related to this study are available in the text of this publication.

## Institutional Animal Use Committee Statement

All experiments involving mice were in compliance with the Association for Research in Vision and Ophthalmology (ARVO) Statement for the Use of Animals in Ophthalmic and Vision Research. All procedures were approved by the Institutional Animal Care and Use Committees (IACUC) of the University of Massachusetts Medical School.
